# The Use of the LanthaScreen TR-FRET CAR Coactivator Assay in the Characterization of Constitutive Androstane Receptor (CAR) Inverse Agonists

**DOI:** 10.3390/s150409265

**Published:** 2015-04-21

**Authors:** Alejandro Carazo, Petr Pávek

**Affiliations:** Department of Pharmacology and Toxicology, Faculty of Pharmacy, Charles University in Prague, Heyrovského 1203, Hradec Kralove CZ500 05, Czech Republic; E-Mail: carazo@faf.cuni.cz

**Keywords:** TR-FRET, terbium, constitutive androstane receptor, pharmacodynamics interactions

## Abstract

The constitutive androstane receptor (CAR) is a critical nuclear receptor in the gene regulation of xenobiotic and endobiotic metabolism. The LanthaScreen^TM^ TR-FRET CAR coactivator assay provides a simple and reliable method to analyze the affinity of a ligand to the human CAR ligand-binding domain (LBD) with no need to use cellular models. This *in silico* assay thus enables the study of direct CAR ligands and the ability to distinguish them from the indirect CAR activators that affect the receptor via the cell signaling-dependent phosphorylation of CAR in cells. For the current paper we characterized the pharmacodynamic interactions of three known CAR inverse agonists/antagonists—PK11195, clotrimazole and androstenol—with the prototype agonist CITCO (6-(4-chlorophenyl)imidazo[2,1-*b*][1,3]thiazole-5-carbaldehyde-*O*-(3,4-dichlorobenzyl)oxime) using the TR-FRET LanthaScreen^TM^ assay. We have confirmed that all three compounds are inverse agonists of human CAR, with IC_50_ 0.51, 0.005, and 0.35 μM, respectively. All the compounds also antagonize the CITCO-mediated activation of CAR, but only clotrimazole was capable to completely reverse the effect of CITCO in the tested concentrations. Thus this method allows identifying not only agonists, but also antagonists and inverse agonists for human CAR as well as to investigate the nature of the pharmacodynamic interactions of CAR ligands.

## 1. Introduction

The LanthaScreen^TM^ Nuclear Receptor Assay is commercially provided by Invitrogen (now a division of Thermo) for several nuclear receptors, including FXR, LXRα, PPARα and PXR. The LanthaScreen^TM^ Constitutive Androstane Receptor Coactivator Assay is a TR-FRET based assay with terbium and fluorescein fluorophores to detect ligands of the Constitutive Androstane Receptor (CAR) nuclear receptor. In contrast to other nuclear receptor LanthaScreen^TM^ assays, which are frequently reported in literature, to our knowledge no paper has been published on the characterization, validation or use of the TR-FRET CAR Coactivator Assay until quite recently, when we published the first article demonstrating the value of the assay in the characterization of indirect CAR ligands [1].

The constitutive androstane receptor (CAR, NR1I3) is a ligand-activated transcription factor with high constitutive activity belonging to the nuclear receptor group NR1I. CAR has been demonstrated to regulate the gene expression of major phase I and phase II xenobiotic-metabolizing enzymes (DMEs) and drug transporters such as cytochrome P450 CYP3A4 and CYP2B6 enzymes, the ABC efflux transporter MRP2 and the conjugation enzyme UGT1A1 [2]. 

CAR has also recently been shown to play important roles in the metabolism of glucose, lipids, and fatty acids as well as in the endobiotic metabolism of bile acids, bilirubin and thyroid hormones. In addition, CAR has been studied as an important factor controlling cell-cycle regulation, apoptosis and cell-cell interaction [3,4]. Human CAR displays unique properties in comparison with other nuclear receptors including high constitutive activity, both direct (ligand-binding domain (LBD)-dependent) and LBD-independent activation, and spontaneous nuclear localization in tumor cell lines. For these reasons, there are currently only few specific and nontoxic ligands of human CAR that can be considered for clinical investigation or for further characterization of human CAR [1,4].

CAR is formed from three domains: a highly conserved DNA-binding domain, a hinge region, and a divergent ligand binding/dimerization/transcriptional activation domain [5]. Both the ligand-binding domain (LBD)-dependent and the independent activation of human CAR have been shown to release CAR from its cytoplasmic tethering complex into the nucleus. Dephosphorylation-induced translocation of CAR to the nucleus has been recently found as the key step toward indirect activation via EGFR-dependent signaling inhibition with phenobarbital [6,7,8].

As indicated earlier, we have recently shown the LanthaScreen^TM^ TR-FRET CAR Coactivator Assay as a suitable method to distinguish indirect CAR activators [1]. The procedure is based on the detection of the energy transfer between terbium bound to the recombinant GSH-tagged human CAR ligand binding domain (LBD) and fluorescein-labeled PGC1α coactivator interacting with CAR LBD. The interaction between CAR LBD and its coactivator PGC1α is triggered by an agonist or released by an inverse agonist or antagonist [1]. The assay provides a simple and reliable method to analyze the affinity to the CAR-LBD with no need to use cell lines, thus eliminating cell signaling [8], cellular transport and metabolism as confounding factors. 

For the current paper we analyzed the pharmacodynamic interactions of the known CAR agonist CITCO (6-(4-chlorophenyl)imidazo[2,1-*b*][1,3]thiazole-5-carbaldehyde-*O*-(3,4-dichlorobenzyl)oxime) [9] with three antagonists/inverse agonists—clotrimazole [10], PK11195 [11] and androstenol [12]—using the LanthaScreen^TM^ CAR Coactivator TR-FRET Assay. Our main goals were to (i) further optimize the protocol of the assay; (ii) apply the method in the study of agonist–antagonist/inverse agonist pharmacodynamic interactions; and (iii) characterize and compare three known inverse agonists/antagonists in the cell-free assay. 

## 2. Experimental Section 

### 2.1. Chemicals 

CITCO **(**6-(4-Chlorophenyl)imidazo[2,1-b][1,3]thiazole-5-carbaldehyde O-(3,4-dichlorobenzyl)oxime) was purchased from BIOMOL Research, Germany. Clotrimazole **(**1-(o-Chlorotrityl)imidazole, 1-(o-Chloro-α,α-diphenylbenzyl)imidazole, 1-[(2-Chlorophenyl)diphenylmethyl]-1H-imidazole), androstenol (5α-androst-16-en-3α-ol) and PK11195 **(**1-(2-Chlorophenyl)-N-methyl-N-(1-methylpropyl)-3-isoquinolinecarboxamide) were purchased from Sigma-Aldrich (St. Louis, MO, USA).

### 2.2. LanthaScreen TR-FRET Coactivator Assay 

**Figure 1:**
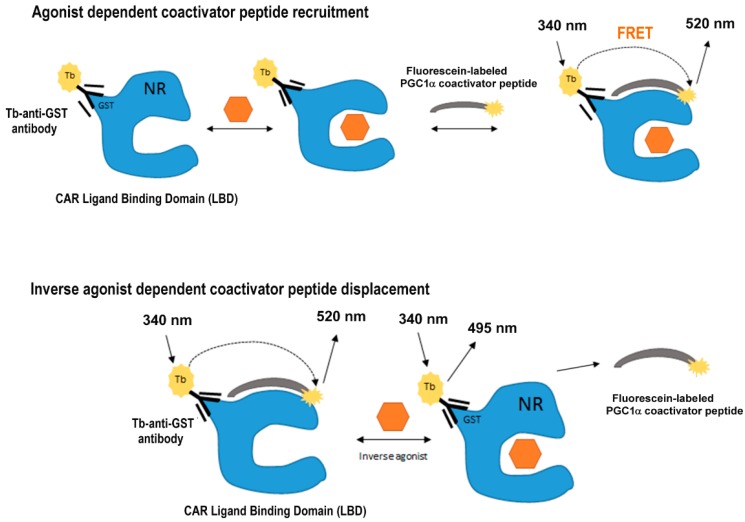
Principle of the TR-FRET (Time-Resolved Fluorescence Resonance Energy Transfer) LanthaSceen^TM^ CAR Coactivator Assay.

The LanthaScreen^TM^TR-FRET CAR Coactivator Binding Assay (Cat. No. PV4836, now produced by Thermo) was used with slight modifications of the manufacturer’s protocol. The assay uses two fluorophores: a terbium-labeled anti-GST antibody interacting with glutathione-S-transferase (GST) tagged human CAR ligand-binding domain (LBD) and a fluorescein-labeled PGC1α coactivator peptide (Figure 1). The interaction of an agonist ligand stimulates the interactions of the components, resulting in energy transfer to the acceptor fluorophore and a FRET (Fluorescence or Förster Resonance Energy Transfer) emission shift from 495 nm to 520 nm when fluorophores are within close proximity to one another. Thus, this energy transfer is detected by an increase in the fluorescence emission of the acceptor (fluorescein) and a decrease in the fluorescence emission of the donor (terbium). To quantify the process, TR-FRET is expressed as a ratio of the intensities of the acceptor and donor fluorophores. 

Since the CAR nuclear receptor has high constitutive activity independent of a ligand, CAR LBD partly interact with the fluorescein-labeled PGC1α coactivator peptide in the absence of ligands. When an inverse agonist or antagonist is bound to CAR LBD, helix 12 of CAR LBD adopts a conformation that precludes fluorescein-labeled PGC1α coactivator peptide binding, and a decrease in TR-FRET is observed.

The assay was performed in a 384-well plate (black round-bottom plates purchased from Corning^TM^) format in 20 µL of reaction mixture. Incubation time was optimized from 1 to 4 h at room temperature and protected from light. The reaction mixture was composed according to the manufacturer’s recommended final concentrations (fluorescein-labeled PGC1 α coactivator 125 nM, Tb-labeled GST antibody 5 nM, CAR LBD GST-tagged protein 5 nM) [13]. 

According to the recommended protocol of the manufacturer, CAR-LBD is added to the solution of test compounds, followed by the addition of a mixture of the fluorescein-coactivator peptide and terbium-labeled anti-GST antibody. In the study we also tested to determine if the order of adding the constituents in the mixture may have an influence on the sensitivity and reproducibility of the assay.

**Figure 2:**
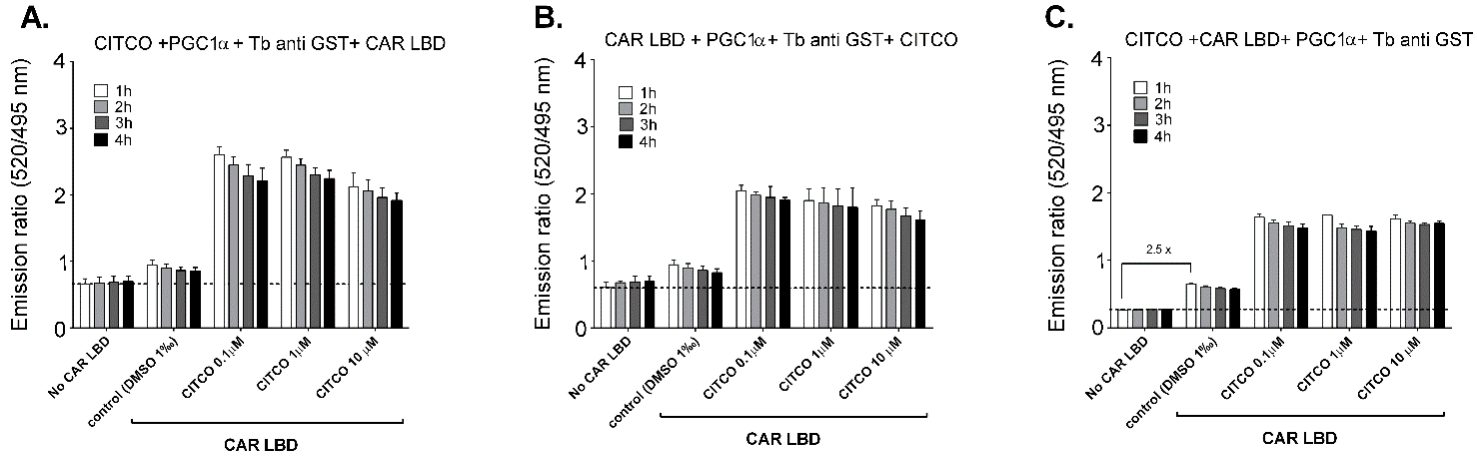
*Optimization of the TR-FRET* (Time-Resolved Fluorescence Resonance Energy Transfer) *LanthaSceen^TM^ Assay.* Our TR-FRET (Time-Resolved Fluorescence Resonance Energy Transfer) experiments were slightly modified to study whether a protocol alteration influenced the response to the CAR agonist. In (**A**), the reaction mixture was composed in the following order: CITCO, the fluorescein-labeled anti-PGC1α coactivator, Tb-labeled GST antibody and CAR LBD; in (**B**), the reaction mixture was composed in the following order: CAR LBD, the fluorescein-labeled anti-PGC1α coactivator, Tb-labeled GST antibody and CITCO; and in (**C**), the standard protocol was followed (the order of CITCO, CAR LBD, the fluorescein-labeled anti-PGC1α coactivator and Tb-labeled GST antibody). The dotted line represents background nonspecific fluorescence in the absence of CAR LBD.

**Figure 3:**
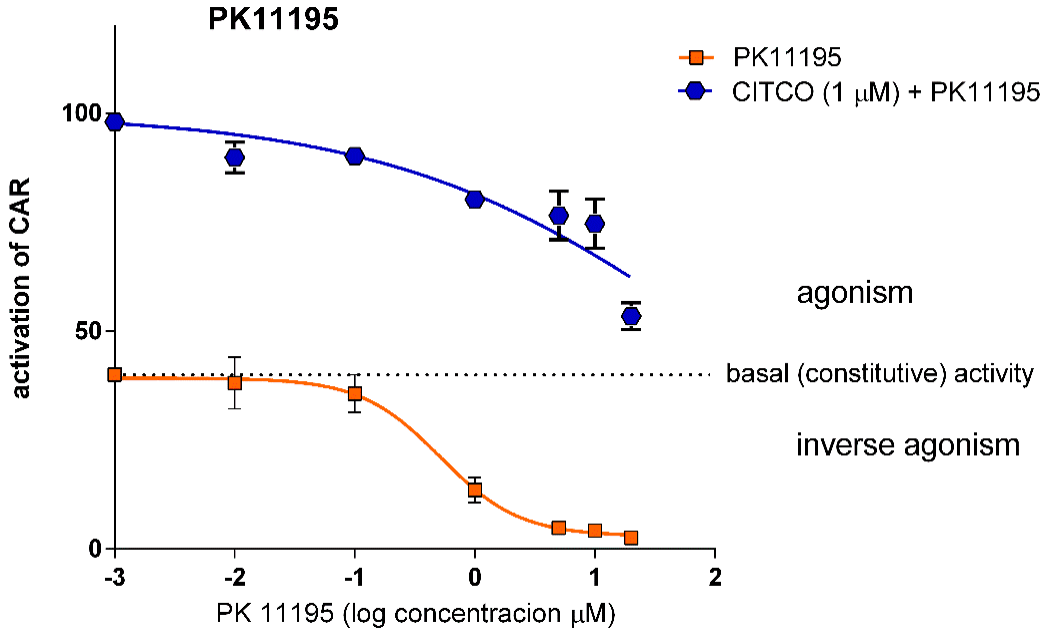
*Effect of PK11195 on CAR LBD activity in the TR-FRET LanthaScreen^TM^ CAR Coactivator Assay.* PK11195 in a serial dilution was tested in inverse agonistic or antagonistic modes together with the prototype CAR agonist CITCO (1 μM concentration) using the TR-FRET assay. Data are presented as the relative activation to background activity (no CAR LBD in the reaction mixture, set to 0%) and to the effect of CITCO (1 μM) set as 100% activation. The dotted line represents the constitutive activity of CAR LBD (vehicle-treated samples). Data are presented as the means and S.D. from three independent experiments (*n* = 3). Dose response curves were fitted using a sigmoidal dose response equation with a variable slope employing the software GraphPad PRISM ver. 6.06.

**Figure 4:**
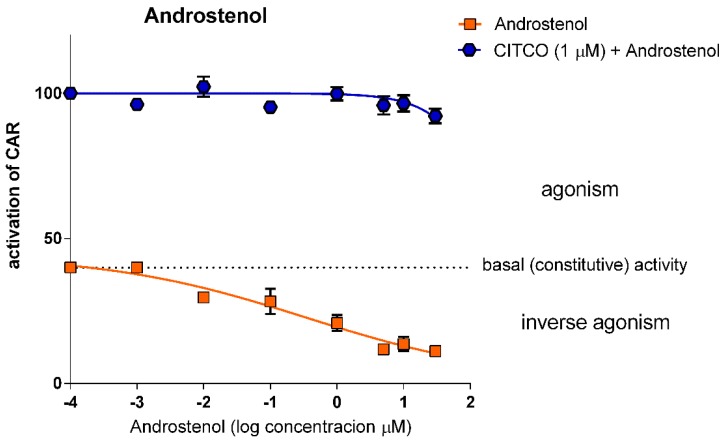
*Effect of androstenol on CAR LBD activity in the TR-FRET LanthaScreen^TM^ CAR Coactivator Assay.* Androstenol in a serial dilution was tested in inverse agonistic or antagonistic modes together with the prototype CAR agonist CITCO (1 μM concentration) using the TR-FRET assay. Data are presented as the relative activation to background activity (set to 0%) and to the effect of CITCO (1 μM) set as 100% activation. The dotted line represents constitutive activity of CAR LBD (vehicle-treated samples). Data are presented as the means and S.D. from three independent experiments (*n* = 3).

**Figure 5:**
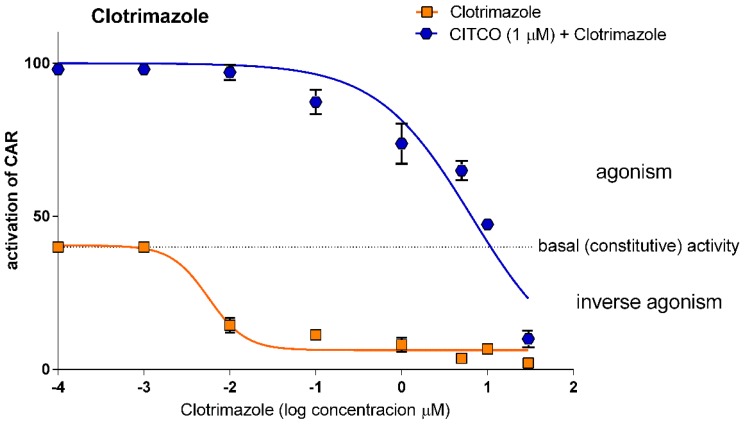
*Effect of clotrimazole on CAR LBD activity in the TR-FRET LanthaScreen^TM^ CAR Coactivator Assay.* Clotrimazole in a serial dilution was tested in inverse agonistic or antagonistic modes together with the prototype CAR agonist CITCO (1 μM concentration) using the TR-FRET assay. Data are presented as the relative activation to background activity (set to 0%) and to the effect of CITCO (1 μM) set as 100% activation. The dotted line represents constitutive activity of CAR LBD (vehicle-treated samples). Data are presented as the means and S.D. from three independent experiments (*n* = 3).

After an incubation period of from 1 to 4 h, TR-FRET fluorescence was measured with a Synergy 2 Biotek plate reader (Bio-tek, Winooski, VT) using an excitation wavelength of 340 nm with filters detecting the fluorescent emission signals of terbium at 495 nm (10 nm bandwidth) and fluorescein at 520 nm (20 nm bandwidth). The terbium was excited using a 340-nm excitation filter with a 30-nm bandwidth and fluorescein was excited with the first emission peak of terbium centered at 495 nm. Energy transfer to fluorescein emission (520 nm) is then measured in the silent region between the first two terbium emission peaks centered at 490 and 545 nm (see Graphical Abstract). In this way, less than 1% of the total Tb^3+^ emission is detected as bleed-through [14]. Delay time was set at 100 μs and integration time 200 μs. Gains set to 100 were the use constant in all the experiments. The 520 nm/495 nm TR-FRET ratio was then calculated. TR-FRET data are presented as 520 nm/495 nm TR-FRET ratio (Figure 2) or as relative activation or inhibition of CAR to control (DMSO vehicle-containing sample) without CAR LBD (set to zero, background) and to CITCO (10 μM)-treated samples (set at 100% activation) (Figure 3, Figure 4 and Figure 5). Values below the baseline value of the sample with CAR LBD and treated with vehicle (0.1% DMSO) represent the inhibition of CAR-PGC1α interaction and suppression of CAR constitutive activity with CAR antagonists/inverse agonists. The values above the baseline represent activation of CAR LBD-PGC1α interaction and agonistic activity of the tested ligand. The experiments were performed at least three times in quadruplicates. Data are presented as the means and S.D. from three independent experiments (*n* = 3). The Z-factor was always higher than 0.5 in our experiments.

In the agonist assay (upper panel), a ligand binds the Constitutive Androstane Receptor (CAR) ligand binding domain (LBD) labeled with the terbium bound anti-GST antibody. Binding of the agonist causes conformational changes of CAR LBD around helix 12 resulting in an increased affinity of the fluorescein-labeled PGC1α coactivator peptide. The close proximity of terbium (donor) and fluorescein (acceptor) causes energy transfer to the fluorescein and TR-FRET in emission at 520 nm after excitation at 340 nm.

In the case of the inverse agonist mode (lower panel), CAR LBD labeled with terbium through the anti-GST antibody partly interacts with the fluorescein-labeled PGC1α coactivator peptide causing constitutive ligand-independent activity of CAR. Binding of an inverse agonist to the CAR LBD produces conformational changes decreasing the affinity of the PGC1α coactivator. The close proximity of the terbium (donor) and fluorescein (acceptor) and the resultant energy transfer TR-FRET is thus disrupted; emission decreases at 520 nm. 

### 2.3. CAR LBD Assembly Assay

The CAR LBD assembly assay was performed according to the protocol we described in our latest report [1]. The CAR LBD assembly assay is based on two hybrid expression constructs encoding C (151–349 aa, helices 3–12, pCAR-C/VP16) and N (103–150 aa, helix 1, pCAR-N/GAL4) terminal parts of human CAR LBD that are co-transfected together with the pGL5-luc luciferase gene reporter plasmid (Promega) containing GAL4 binding sites. When the CAR LBD interacts with a ligand (both agonist and antagonist), connection of the helix 1 to CAR LBD helices 3–12 promotes firefly luciferase activation. Thus, the assay monitor interaction of CAR LBD with ligands rather than its activation or deactivation. Experiments have been done in HepG2 cells with CITCO (1 µM) as an agonist and with serial dilutions (range 0.1–30 µM) of CAR antagonists clotrimazole, PK11195 and androstenol, respectively. IC_50_ has been calculated for each compound from at least five data points.

### 2.4. Data Analysis

Dose-response curves were generated by plotting the emission TR-FRET ratio *vs*. the log of a ligand (in μM). To determine the half maximal inhibitory concentration **(**IC_50_) value, the data were fitted using an equation for a sigmoidal dose response inhibition (with a varying slope) using the software GraphPad™ PRISM version 6.05. Z-factors were calculated using the method of Zhang *et al*. [15].

## 3. Results and Discussion

### 3.1. Results 

#### 3.1.1. Optimization of TR-FRET Mixture Composition and Incubation Times

In the first experiments, we determined whether modifications in the procedure provided by the manufacturer would have an influence on the sensitivity of the assay for an agonist as well as on the reduction of non-specific background. We altered the order in which the reagents were added to the reaction mixture and compared the results with the signals obtained with the standard procedure. One of the modifications was to add the ligand (CITCO), then a fluorescein-labeled PGC1α coactivator and terbium-labeled anti GSH antibody and finally the CAR-LBD solution. Another modification consisted of adding CAR-LBD and fluorescein-/terbium-labeled reagents solutions before the agonist. As shown in Figure 2, both modifications proved to be effective (high fold-activation by the ligand) but the results showed less sensitivity than the signals obtained by the standard procedure (the fold activation by CITCO 10 μM was 2.26; 1.94; and 2.56, respectively, compared to vehicle control). We also found that the standard protocol recommended by manufacturer results in a relatively lower background (the sample without CAR LBD, 2.5-fold lower) in comparison with the control sample with vehicle (DMSO 0.1%), CAR LBD, Tb-labeled anti-GSH antibody and fluorescein-labeled PGC1α coactivator (Figure 2C).

Secondly, we tested to see if incubation time had an effect on the outcomes of the experiments. We observed no significant effect of incubation time in all three experimental setups (Figure 2), although a slightly higher FRET ratio was observed for the incubation time of 1-h. These data confirm published results [13].

Thus these results confirm that the recommended experimental protocol and 1-h incubation time are optimal for further experiments.

#### 3.1.2. PK11195 Shows Competitive Inhibition for CAR 

PK1195 **(**1-(2-chlorophenyl)-N-methyl-N-(1-methylpropyl)-3-isoquinolinecarboxamide) is a known antagonist for human CAR, acting as a direct ligand for the receptor [11]. PK11195 has been shown to act as an antagonist of CITCO as well as an inverse agonist of constitutive activity in vehicle-treated samples in gene reporter assays and in RT-PCR experiments [11,16]. In our experiments, we confirmed that PK11195 is an inverse agonist with IC_50_ 0.51 μM (Table 1) to reduce CAR constitutive activity (Figure 3). We also observed concentration-dependent antagonistic competition of PK11195 with CITCO for the CAR LBD, as shown in Figure 3. Significantly, PK11195 at the concentration of 20 μM almost completely abolished the constitutive (CITCO-independent) interaction of CAR LBD with PGC1α (Figure 3).

In the experiments agonist CITCO was always added into TR-FRET reaction mixture before PK11195. But no significant difference was observed in the TR-FRET fluorescence signals when PK11195 was added before CITCO (*unpublished data*).

#### 3.1.3. Androstenol does not Show Significant Competitive Inhibition for CAR LBD

Unlike PK11195, androstane metabolites are weak inverse agonists of human CAR [12]. Androstenol (5α-androst-16-en-3α-ol) did not show any significant inhibition for CITCO-mediated CAR LBD interaction with PGC1α in our TR-FRET assays (Figure 4). The consistent antagonistic effect of androstenol on CITCO was only observed at the 30-μM concentration (Figure 4). In agreement with previous reports, androstenol decreased constitutive activity of CAR with IC_50_ 0.345 μM (Table 1) in our TR-FRET experiments, a result which is in accordance with published data [13]. Again, as in the case of PK11195, no statistical difference was observed between the protocols when CITCO was added before androstenol or the other way round. 

#### 3.1.4. Clotrimazole is a Potent Antagonist of Human CAR 

Clotrimazole, an antifungal azole drug, has been reported as a human CAR antagonist [10]. In our work a significant inhibition of the CAR LBD constitutive activity was demonstrated already in concentrations of 0.01 μM and 10 μM clotrimazole almost completely inhibited the interaction of CAR LBD with the PGC1α coactivator in the TR-FRET assay (Figure 5). The IC_50_ for clotrimazole was 0.005 μM (Table 1), which indicates high affinity to CAR LBD at low concentrations of clotrimazole. When combined with CITCO (either 1 or 10 μM), both concentration-dependent curves show the same profile (Figure 5) (*unpublished data for 10 μM CITCO*). Notably, at the 30-μM concentration, we observed that clotrimazole completely abrogated the effect of CITCO on CAR LBD interaction with PGC1α as well as further decreased the interaction below the baseline of CAR constitutive activity (Figure 5). These data demonstrate that clotrimazole is a highly potent antagonist and inverse agonist of human CAR that is able to completely inhibit CAR coactivation with PGC1α in low micromolar concentrations.

These data indicate that the tested inverse agonists/antagonists inhibit CAR coactivation with PGC1α in the TR-FRET assay in the following order: clotrimazole > PK11195 > androstenol. Clotrimazole was the only compound to reverse the effect of CITCO in the tested concentrations. Clotrimazole and PK111195 at the concentrations of 20 and 30 μM abolished constitutive activity of CAR and completely disrupted the interaction of CAR LBD with PGC1α (Figure 3 and Figure 5), yielding background FRET ratio activities.

#### 3.1.5. Clotrimazole is a Potent Antagonist of Human CAR 

To confirm our TR-FRET CAR Coactivator assay data, CAR LBD assembly assay has been employed/performed. The assay directly monitors interaction of a ligand with the CAR LBD producing a firefly luciferase gene reporter vector activation. We observed similar IC50s for clotrimazole and PK11195 antagonist in the assay in comparison with TR-FRET CAR coactivation assay (Table 1). On the other hand, androstenol exerted a weaker inhibition of the activity of CITCO and the data we obtained were not reliable to obtain kinetic data of the curve.

**Table 1:** Parameters of tested compounds to interact with human CAR LBD.

	IC_50_ (μM)(95% Conf. Interv.)/R Square		
	TR- FRET CAR Assay		CAR LBD Assembly Assay
Compound	No CITCO	CITCO 1 μM	CITCO 1 μM
PK11195	0.51 (0.36–0.72) 0.998	93.63 (9.24–948.3) 0.858	56.61 (36.68–87.37) 0.905
Androstenol	0.345 (0.01–670.8) 0.968	312.6 (152.8–676.8) 0.318	N.A.
Clotrimazole	0.005 (0.0008–0.039) 0.968	6.15 (3.42–12.41) 0.932	11.37 (2.59–49.71) 0.865

N.A.—not available, not reliable fitting in the concentration range.

### 3.2. Discussion 

The use of the TR-FRET assay to analyze the ligands of a nuclear receptor was first reported by Parks *et al*. [17], who developed an europium-based assay with biotinylated Farnesoid X receptor (FXR) LBD labeled with streptavidin-conjugated allophycocyanin (APC) dye and the 5′-biotinyled SRC1 coactivator peptide fragment (AA 676 to 700) labeled with streptavidin-conjugated europium chelate. The first TR-FRET europium-based assay to detect mice and human CAR nuclear receptor ligands was described by Moore *et al*. In this assay, biotinylated mouse and human CAR LBDs labeled with the steptavidin-conjugated fluorophore APC dye interacted with the peptide L*XX*LL motif of SRC-1 coactivator (AA 676–700) labeled with europium chelate [9,10]; this process was patented in 2011 [18].

In contrast to standard FRET assays, TR-FRET assays use a long-lifetime lanthanide chelate as the donor fluorophores [14]. Lanthanide chelates have extremely-long excited-state lifetime, on the order of a millisecond when the molecule spends in the excited state after accepting a photon [14]. The delay time of TR-FRET measurement is usually 50 to 100 microseconds after a flashlamp excitation. Therefore, TR-FRET eliminates interference from autofluorescent compounds as well as from scattered light, since the interference is in the nanosecond timescale. The most common lanthanides used in TR-FRET assays are terbium (Tb^3+^) and europium (Eu^3+^). Terbium-based TR-FRET assays can be conducted with common, cheap and easy-to-work fluorophores, such as fluorescein, as the acceptor in fluorescein-labeled molecules; this is in stark contrast to europium-based systems, which employ APC as the acceptor in biotinylated molecules. Thus the main advantage of the TR-FRET assay is overcoming interference from compound autofluorescence and light scatter from precipitated compounds. In addition, considering FRET as a ratio of the intensities of the acceptor and donor fluorophores, differences in assay volumes between wells can be eliminated and quenching effects due to colored compounds can be corrected using this approach. 

The LanthaScreen™ time-resolved Förster resonance energy transfer (TR-FRET) technology was introduced by Invitrogen (now a division of Thermo) in 2007. The assay facilitates the discovery and evaluation of compounds that bind to human CAR LBD based on the recruitment or displacement of the coactivator-based fluorescein-labeled peptide PGC1α. The interaction of CAR LBD with PGC1α is controlled by a conformational change in the CAR receptor LBD around helix 12 that takes place upon ligand binding. Either the constitutive association of CAR LBD with a fluorescein-labeled coactivator peptide may be disrupted by inverse agonists, or the interaction between the receptor and fluorescein-labeled PGC1α coactivator peptide is augmented by agonists. This interaction is detected by monitoring the FRET signal between a terbium-labeled anti-GST antibody bound to CAR LBD and the fluorescein-labeled peptide PGC1α. 

As far as we know, ours is the only research group reporting on the LanthaSceen^TM^ CAR Coactivator Assay at the present time. In another recent publication, we demonstrated the applicability of the assay for distinguishing indirect CAR activators among the natural flavonoids, which activate CAR via posttranslational modification [1]. For the current work, using the TR-FRET LanthaSceen^TM^ assay we studied three well-known inverse agonists/antagonists of human CAR. We found that clotrimazole is the most potent antagonist and inverse agonist of human CAR, followed by PK11195 and androstenol (Table 1). Clotrimazole was the only compound to reverse the effect of CITCO in the tested concentrations. Another interesting finding of the study is that at the concentration of 30 μM clotrimazole and PK111195 abolish the constitutive activity of CAR and completely disrupt the interaction of CAR LBD with PGC1α (Figure 3 and Figure 5). 

The assembly assay method provides reliable information about the affinity of a studied substance to bind to CAR-LBD. In this regard, clotrimazole showed the most significant activity to revert the CAR activation by CITCO 1µM in a dose-dependent manner (IC_50_ = 11.4 ± 1.7 µM), while PK11195 showed less potent inhibitory effect (IC_50_ = 56.6 ± 1.2 µM). Androstenol displayed the weakest inhibition even in the highest studied concentration (30 µM) against CITCO 1 µM. These data are in accordance to the data obtained by the TR-FRET technology. Thus, both assays prove to be sensible, reliable and interchangeable methods to study ligand interaction and competition for CAR-LBD.

## 4. Conclusions

In this article, we communicate our results demonstrating that the TF-FRET CAR coactivator assay is a suitable, rapid (2 h), and convenient (mix-and-measure mode) tool for the interaction testing of human CAR and its ligands without the confounding effect of cellular signaling and cell-dependent posttranslational modification of CAR. Further, the assay can be adapted for a high-throughput screening format (384-well plate). Our current studies also show for the first time the pharmacodynamic interactions of a CAR agonist and an inverse agonist/antagonist *in silico*. 
